# A Novel Vaping Machine Dedicated to Fully Controlling the Generation of E-Cigarette Emissions

**DOI:** 10.3390/ijerph14101225

**Published:** 2017-10-14

**Authors:** Sébastien Soulet, Charly Pairaud, Hélène Lalo

**Affiliations:** Laboratoire Français du E-Liquide (LFEL), 218 avenue du Haut-Levêque, 33600 Pessac, France; charly.pairaud@lfel.fr

**Keywords:** electronic cigarette, vaping machine, emission generation, standardization

## Abstract

The accurate study of aerosol composition and nicotine release by electronic cigarettes is a major issue. In order to fully and correctly characterize aerosol, emission generation has to be completely mastered. This study describes an original vaping machine named Universal System for Analysis of Vaping (U-SAV), dedicated to vaping product study, enabling the control and real-time monitoring of applied flow rate and power. Repeatability and stability of the machine are demonstrated on flow rate, power regulation and e-liquid consumption. The emission protocol used to characterize the vaping machine is based on the AFNOR-XP-D90-300-3 standard (15 W power, 1 Ω atomizer resistance, 100 puffs collected per session, 1.1 L/min airflow rate). Each of the parameters has been verified with two standardized liquids by studying mass variations, power regulation and flow rate stability. U-SAV presents the required and necessary stability for the full control of emission generation. The U-SAV is recognised by the French association for standardization (AFNOR), European Committee for Standardization (CEN) and International Standards Organisation (ISO) as a vaping machine. It can be used to highlight the influence of the e-liquid composition, user behaviour and nature of the device, on the e-liquid consumption and aerosol composition.

## 1. Introduction

The use of electronic cigarettes (ECs) has grown rapidly as a safer alternative for nicotine consumption than tobacco cigarettes [1]. The principle of an EC is an electrically powered heating element that generates an aerosol from an e-liquid which generally contains a solution of propylene glycol (PG), glycerol (VG), flavours and nicotine. Globally, the design remains the same for all ECs: an atomiser connected to a battery, with the possibility to vary voltage, and power to control temperature. An EC is made up of a tank containing the e-liquid and an atomizer head (a metal coil wrapped around a wick usually composed of cotton). Depending on the models, the users can have a disposable cartridge (combining liquid and atomizer head), a cartomizer to be refilled (atomizer head already included) or a rebuildable one, allowing users to build their own atomizer head (choice of wick and resistor: metal, value, geometry). The e-liquid present in the tank moves by capillarity into the wick. When a user activates the EC, the resistor heats up by transforming electrical energy from the battery into thermal energy, using the Joule effect [2]. This temperature elevation heats the wick and evaporates the e-liquid. This implies the local drying up of the wick (where it is surrounded by the resistor) and the displacement of e-liquid by capillarity. Airflow through the atomizer is induced by the user’s inhalation, and this contributes to the thermal balance of the wire. Each of the previously quoted parameters can influence the composition of the aerosol [3]. Firstly in France (French association for standardization (AFNOR)) [4] then worldwide (European Committee for Standardization (CEN)) and International Standards Organisation (ISO)), working groups have been created in order to define standards for emission generation. Indeed, aerosol composition and e-liquid consumption, as well as nicotine release, are completely dependent upon the conditions of aerosol generation [5]. In order to obtain comparative results, emissions have to be generated in the exact same way for all smoking or vaping machines. That means all influential physical parameters have to be carefully monitored.

Currently, investigations about the aerosol composition, nature and quantity of molecules [6,7,8,9,10,11], size of particles [12,13], toxicity (in humans [14] and in vitro [15]), are performed with smoking machines adapted to vaping products. In these cases, commercial batteries are used and important variations can occur due to the lack of repeatability of the device and the inaccurate measurement of the resistance value during the experiment. Therefore, questions remain about the variability of nicotine release [16] or the proper measurement of aldehydes rate in the aerosol [9]. In specific cases, we could not say whether the results came directly from electronic cigarettes or from a smoking machine that does not enable to fully control the device. 

To illustrate this point, a battery can discharge and have limited power delivery, the level of power delivery may vary from one puff to the next or it may provide a false reading of applied power. Additionally, the resistor can be defective and can easily overheat during the manipulation. In such cases, the operator cannot observe any of these crucial problems with the smoking machines currently used for such studies.

**Figure 1:**
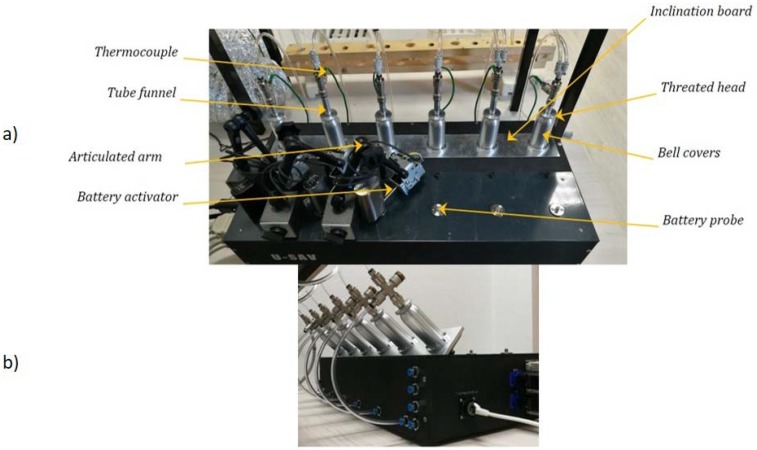
Picture of the Universal System for Analysis of Vaping (U-SAV) machine. U-SAV enables (**a**) to plug wide range of atomisers on the market as well as batteries and trigger them, and (**b**) to tilt the atomiser from 0° to 90°.

The current study presents a novel vaping machine specifically designed for ECs that enables the control and monitoring of relevant physical parameters during aerosol generation. This machine, named Universal System for Analysis of Vaping (U-SAV, Figure 1), is characterized by the following features:

- Generation and control of the electrical energy supplied to the resistor;
- Measurement of the electrical parameters when using an external battery device;
- Control of a large range of airflow rate;
- Calculation of resistor value;
- Ability to control the electrical energy delivery, either by voltage or by power regulation, using an internal (built-in) generator; Generation of different flow profiles (square, tooth saw, sinusoidal).

All these parameters are monitored in real time and the operator has the possibility to watch them and notice any abnormality. In this publication, we are going to demonstrate: the stability of flow rate and electrical energy supplied, as well as their repeatability, by monitoring their value and measuring mass variation of the standardized liquids used. 

The normative reference used in this study is the French AFNOR standard XP-D90-300 part 3 [4] and the ISO 20768 standard (currently in the final validation process by ISO). The protocol applied for emission generation and standardized liquids A and B, defined in AFNOR standard [4], is described below.

## 2. Materials and Methods 

### 2.1. Vaping Machine Configuration

U-SAV has the distinctive feature to generate and control the electrical energy delivered to the atomiser [17]. Its design allows the connection of atomiser with airflow entries on the top, bottom or sides. U-SAV has 6 vaporisation lines, separated in 2 triplicates. Each of the 6 lines can have different settings for power or flow rate applied, as well as different modes of electrical energy supplied or flow profile. The operator can choose between three different flow profiles (square, tooth saw, sinusoidal) and different modes:

- Voltage: a constant voltage is applied during the whole manipulation,
- Power: the resistor value is calculated every 150 ms by measuring current and voltage and the power supplied is regulated according to the evolutions of resistor value.

The functioning cycle (puff number and duration) is common for all 3 lines in a triplicate.

For each triplicate, airflow can be delivered in either suction or air-inlet mode (drawing air after the atomizer or forcing air before the atomizer). U-SAV is also equipped with 6 screw threads (510 threads), enabling the use of almost all commercially available EC battery devices. U-SAV can record in real-time all the electronic parameters of the battery and can determine any difference between the power setting and the true power delivery. 

In this study, the AFNOR protocol for the generation of emission is used. The manipulation is composed of 100 puffs divided into 5 series of 20 puffs. Each puffing cycle lasts 30 s, which include 3 s of aerosolisation and 27 s of rest. The inter-series duration is 300 s. The flow rate is programmed at 1.11 L/min (18.3 mL/s). The inclination of the atomisers is set at 45° during a series of 20 puffs and back to 0°, within 10 s, during the inter-series interval.

### 2.2. Vaping Device

The vaping device used for the manipulations is the Cubis atomiser (Joyetech, Shenzhen, China) with a BF SS316 atomizer head and a 1 Ω resistor. It is composed of a 0.25 mm diameter wire with an overall length of 12 cm. The wire is composed of two different materials. The central part is a stainless steel 316 L wire that is 6 cm in length and is connected to a 3 cm non-resistive wire on both sides. These two pieces are used to fix the wire to the atomiser bracket. This resistor is rolled around a 2.5 mm diameter wick.

### 2.3. Energy Supplier

The internal energy supply of U-SAV is used and compared with two commercial EC batteries: the Evic VTwo (Joyetech, Shenzhen, China) and the Elfin DNA40 (S-body, Shenzhen, China). The atomizer manufacturer provides a range of usable power between 10 and 25 W; the chosen power setting is 15 W.

### 2.4. E-Liquids

Referring to the AFNOR standard XP-D90-300 part 3, two standardized e-liquids are prepared. The aim is to characterize the machine and the atomiser. These two liquids, named liquid A and liquid B, have the same level of nicotine (1/100 g, ALCHEM ≥ 99.2%), food-grade ethanol (1/100 g) and flavour content (10/100 g) but different concentrations of water (A: 1/100 g; B: 2/100 g), propylene glycol (PG, A: 63/100 g; B: 38/100 g, BRENNTAG ≥ 99.8%), and glycerol (VG, A: 24/100 g; B: 38/100 g, AMI CHIMIE 99.5%). The flavouring preparation is composed of vanillin (1 g/100 g, SIGMA-ALDRICH ≥ 97%), isoamyl alcohol (2/100 g, SIGMA-ALDRICH ≥ 98%), 2-methylbutyric acid (1/100 g, SIGMA-ALDRICH ≥ 98%), and PG (96/100 g). 

### 2.5. Characterization of a Vaping Machine

Referring to the ISO 20768 and AFNOR XP D90-300-3 standards, a vaping machine should meet the following conditions for emission generation:

- The puff profile should have a rectangular shape, measured at the port of the machine, with a variation tolerance of 10%.
- The variation of the mass of the e-liquid consumed between different 100-puff sessions has to be less than 25% (calculated based on average variation of 9 sessions for each of the two standardized e-liquids)

In order to meet the characterisation criteria of each standardized e-liquids (A and B), the six lines of U-SAV have been configured in the same way. Four experiments have been conducted:

- Applying 15 W with the regulation of the built-in generator, using e-liquid A
- Applying 15 W with the regulation of the built-in generator, using e-liquid B
- Applying 3.87 V with the built-in generator but without any regulation, using e-liquid A
- Applying 3.87 V with the built-in generator but without any regulation, using e-liquid B

Each experiment has been carried out 6 times for each of the 6 lines, leading to 36 experiments to evaluate stability and reproducibility of power, flow rate and mass of e-liquid aerosolized. To control the last parameter, the atomiser is weighted before and after the experiment. The data for each experiment have been compiled and presented in reference to two configurations: stability on one vaporization line during one puff, and stability and repeatability of each vaporization line during the whole experiment (100 puffs). Aside from the experiments on mass variation (see Section 3.4 and Section 3.5), results shown do not differentiate between the two standardized e-liquids.

A final experiment was conducted to compare battery efficiency with the U-SAV built-in generator. For that purpose, we chose two batteries from different commercial references and used them for the generation of emission referring to the AFNOR standard protocol. We plugged three batteries from each commercial reference on three different lines of U-SAV. Then we applied the AFNOR standard protocol to each line and tracked the value of resistance and true power applied by the batteries in real-time. The mass of e-liquid consumed was measured by weighting the atomiser before and after each session, in order to compare the variation of aerosolized e-liquid with commercial batteries and with the U-SAV built-in generator. Each experiment was performed twice, leading to 6 different tests by battery.

## 3. Results

### 3.1. Flow Rate Regulations (Standardised E-Liquids)

**Figure 2:**
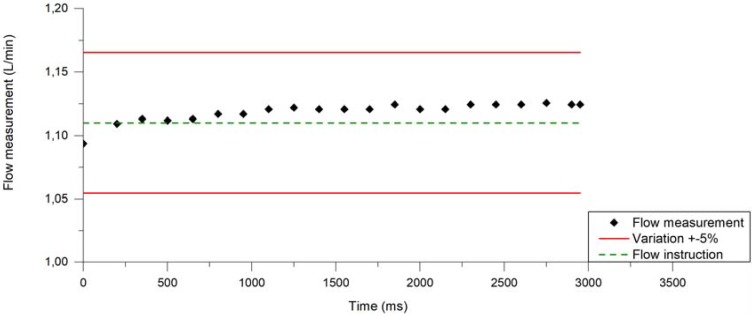
Flow profile puffs generated on U-SAV (dark dots) at 1.11 L/min airflow rate. Red lines represent ±5%.

Figure 2 illustrates the typical flow rate profile during one puff. Through a system of solenoid valves that accurately guide airflow to the EC only when the aerosolisation process occurs, the flow profile is square shaped with less than 10% variation, as required in AFNOR and ISO standards. Flow rate is stabilized at the set value in less than 50 ms and the flow rate variation (RSD) is <5%.

**Figure 3:**
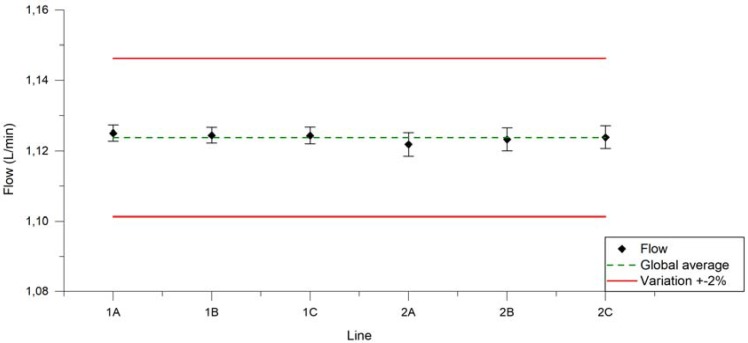
Average air flow rate applied from each U-SAV line over 100 puffs during 24 experiments (dark dots). Red lines represent ±2%. Green line represents the average from all U-SAV lines.

Figure 3 displays the average and standard deviation of airflow rate for each U-SAV line when U-SAV is programmed for a rate of 1.11 L/min. The relative standard deviation (RSD) was <1% for all lines.

### 3.2. Power Regulation

**Figure 4:**
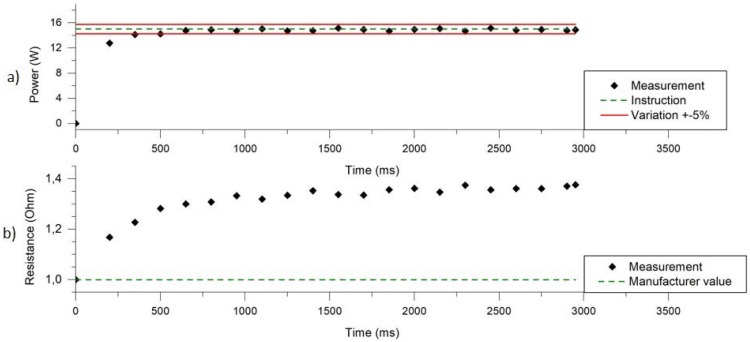
Power applied to the resistor (**a**) and resistor value (**b**) during a typical puff.

Figure 4a,b show, respectively, a typical power and resistor profile obtained with the U-SAV. Two main phases can be observed for both of these curves. The first one is transient and corresponds to a fast increase in resistance value and power delivery at the beginning of the puff (<0.25 s) and the second is a threshold value. The two curves follow the same trend since the power value has been regulated with reference to the resistor value, calculated from measurements of voltage and current. As the resistor value is linked to its temperature (stainless steel wire has a high temperature coefficient of resistance), it becomes stable only when the temperature reaches a plateau value (around 1.35 Ω), which occurs after 500 ms. However, between the transient slope and the complete stabilisation of the temperature, resistor value does not change substantially (approximately 0.15 Ω), which makes it possible to regulate power at 15 W (which was the intended setting) during more than 90% of the puff duration, with a variation lower than 5%. 

**Figure 5:**
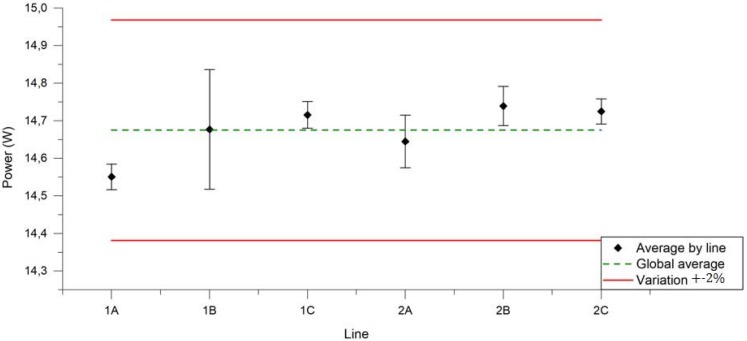
Average power applied from each U-SAV line over 100 puffs during 24 manipulations (dark dots). Red lines represent ±2%. Green line represents the average from all U-SAV lines.

Figure 5 displays separately for each U-SAV line the average power applied over 100 puffs. The power applied is close to 14.7 W during the whole manipulation for each vaporisation line and low RSD is observed (<2%). 

### 3.3. Voltage Regulation

**Figure 6:**
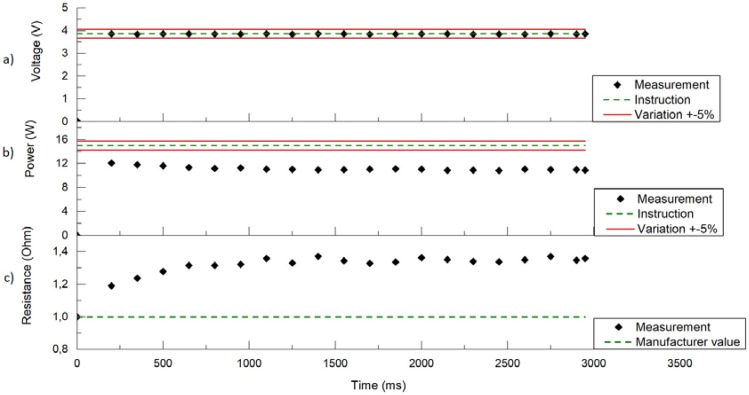
Voltage applied to the resistor (**a**), corresponding power applied (**b**) and resistor value (**c**) during a typical puff.

Another option of the U-SAV allows for controlling the electrical energy delivered through voltage regulation. Figure 6 displays the voltage (a), power (b) and resistor value (c) during one puff in this mode. The voltage was set at 3.87 V, and variation is <5%. However, the corresponding power applied appears to stabilise at 11.8 W when the resistor value reaches a plateau (around 1.35 Ω), similar to the previous experiment. 

**Figure 7:**
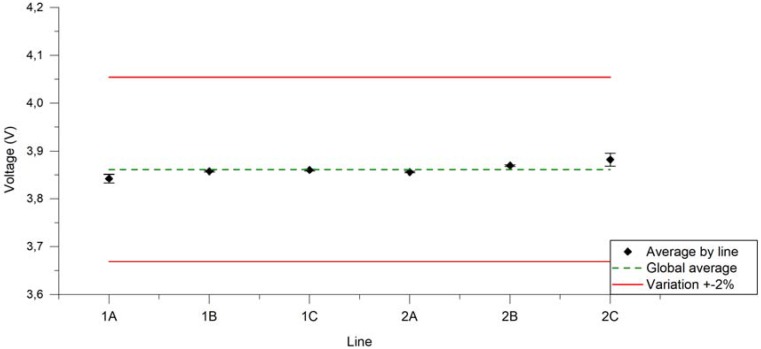
Average voltage applied from each U-SAV line over 100 puffs during 24 manipulations (dark dots). Red lines represent ±2%. Green line represents the average from all U-SAV lines.

Figure 7 represents, the average voltage applied from each U-SAV line over 100 puffs during 24 manipulations. Minimal differences between lines and a 2% variation from the programmed value are observed.

### 3.4. Repeatability on the Mass of Aerosolized E-Liquid 

The results of the average consumption of liquids A and B, as well as their deviation, during an experiment with U-SAV programed in power mode, are presented in Table 1. For both liquids, the RSD is lower than 10%, showing a good repeatability of the aerosolisation process in U-SAV. The average consumption of liquid A is higher than the one for liquid B by 1.30 mg/puff.

The results of average consumption of liquids A and B during the experiment in voltage mode are presented in Table 2. While more substantial than in power mode, RSDs are still lower than AFNOR recommendations defined at 25%. 

It should be noted that liquid consumption per puff is lower for voltage than for power modes. The amount of liquid aerosolised decreases by 30% (for liquid A) to 40% (for liquid B) when constant voltage is applied to the atomiser. 

### 3.5. Batteries Comparison

In order to compare the accuracy and stability of U-SAV’s built-in energy delivery system with commercial batteries, we carried out the AFNOR standard protocol with two different commercial batteries. Three batteries for each commercial reference were plugged into U-SAV. Each experiment was performed twice.

As shown in Table 3, for the same power setting (15 Watts), the true average power applied is lower for the two batteries tested than for U-SAV. Also, the RSD is lower for U-SAV (0.44%) compared to the commercial batteries (1.65% and 2.84%). Elfin DNA 40 batteries (S-body, Shenzhen, China) show better stability and repeatability compared to the Evic VTwo batteries (Joyetech, Shenzhen, China). Additionally, both devices applied lower average power compared to the U-SAV, but the difference was only 1.90% for the Elfin DNA40 and 5.65% for the EvicTwo.

A difference between the U-SAV’s built-in energy delivery system and commercial batteries in the mass of aerosolized e-liquid is also observed (Table 4). The average consumption is lower for both commercial batteries than for U-SAV, which is expected considering the observations on power regulation and delivery. 

## 4. Discussion

### 4.1. Power Regulation

We have observed the resistor value of the atomizer varies during a puff (Figure 4b). The transient slope observed during the first 250 ms is due to a fast temperature elevation that increases the resistor value. The plateau reached after 500 ms is the consequence of a thermal balance between the energy delivered by the battery and the energy collected by the surrounding environment. 

The behaviour of a wire in a convective exchange between the environment and an electrical source has a thermal balance that is shown in Equation (1) [18,19,20]:

(1) $$V_{r}\rho_{r}C_{p_{r}}\frac{\partial T_{r}\left(t\right)}{\partial t}=P-{\mathrm{hS}}_{r}\left(T_{r}\left(t\right)-T_{\mathrm{out}}\right),$$

with:

V_r_ Volume of the resistor (V_r_ = length × section = (0.06 × 0.000125^2^ × π) m^3^)
ρ_r_ Metal density (Stainless steel = 7960 kg/m^3^)
C_p_r__ Metal heat capacity (Stainless steel = 502 J/(K.kg))
T_r_ Temperature of the resistor
P Power delivered (P = 15 W)
h External heat coefficient
S_r_ External surface of the resistor
T_out_ Temperature of external environment

This simple model (1) has two main solutions:

- During the transient response, Equation (1) can be expressed as a straight line:
  (2) $$V_{r}\rho_{r}C_{p_{r}}\frac{\partial T_{r}\left(t\right)}{\partial t}=P,$$
-When the saturation stage is achieved, the function reaches a limit that is characterised by a balance between supplied and diffused energy. This is demonstrated as constant temperature inside the resistor, which is expressed by:
  (3) $$P-{\mathrm{hS}}_{r}\left(T_{r}\left(t\right)-T_{\mathrm{out}}\right)=0,$$

Equation (2) determines the rise of temperature in the resistor. Herein, the temperature rises at a rate of 1275 °C/s. We have built our hypothesis on the fact that the temperature of vaporization of the liquid limits the temperature of the resistor (PG: 188 °C, VG: 290 °C [21]). Thus, referring to Equation (2), the resistor will reach a thermal balance around 0.25 s. This is confirmed by the resistor profile shown on Figure 4b. Indeed, the transient slope occurs during the initial 250 ms of the puff. During this interval, the resistor value increases by 20%. Then, the resistor enters in a saturation stage (Equation (3)) and begins to stabilize until it reaches the value of 1.35 Ω at 1000 ms. At this stage, the resistor value increases by 12% during 750 ms. 

Based on continuous (every 150 ms) resistor measurements and the power set on the U-SAV, a new voltage value is calculated and is applied in order to regulate the electrical power and maintain it at the value programmed by the operator. This is particularly critical during the transient response since the resistance value changes considerably and this is the reason why the power applied during this phase is lower than the power set during the transient response phase. However, the power regulation is very accurate when the resistor reaches a constant temperature. The stability and repeatability of the power delivery makes it possible to have repeatable conditions for aerosol generation.

### 4.2. Voltage Regulation

In voltage mode, the programmed voltage is calculated using the manufacturer resistor value. However, U-SAV does not perform any voltage adjustments in relation to the resistance value change during the puff. As displayed in Figure 4b, the resistance value changes from 1 Ω to 1.35 Ω during the puff. Thus, the applied power changes from to 15.0 W (for a resistance value of 1 Ω) to 11.1 W (for a resistance value of 1.35 Ω). This represents a significant deviation from the expected value (15 W) and explains the difference between set and true (measured) power as shown in Figure 6b. 

Resistor profiles, from power mode (Figure 4b) and voltage mode (Figure 6c), are similar, and shows that the power difference between the two modes does not have any influence on the temperature reached by the resistor. However, liquid consumption between the two different modes was different. A decrease of only 4 W on a 3 s puff results in a 30 to 40% reduction in the mass of liquid aerosolized. This experiment proves that it is extremely important to have full monitoring of the aerosolization process. Likewise, it is essential to have the possibility to alter the voltage supply to the atomiser in order to have a constant and accurate power delivery to the resistor. These observations have implications in the regulatory setting when comparing efficiency or nicotine release from different devices. 

### 4.3. Batteries Comparison

Each of the two batteries from different commercial brands was tested for stability of the power supplied and repeatability of the e-liquid aerosolised. The RSD of the power applied is higher for the commercial batteries than for the U-SAV but it is still lower than 5% and should thus be considered acceptable. However, the observed difference has a direct impact on the average consumption of e-liquid. The Evic battery aerosolises 15% less e-liquid compared to the U-SAV. This should be taken into consideration when evaluating device efficiency or comparing findings between different laboratories. Despite consistent delivery of power throughout the experimental session (100 puffs in this study), still there was a substantial difference between the desired and the true power delivery. This is a major issue in the study of vaping products. A battery can present a good stability for energy supplied and mass of aerosolized e-liquid but have a relevant deviation in comparison with the instructions asked. 

This has to be taken into consideration for the next review of AFNOR standards and for the upcoming CEN and ISO standards. It is important for any regulation to set a maximum variability for the power delivered by each device, with 5% RSD being a reasonable requirement. 

### 4.4. Flow Rate Control

The U-SAV vaping machine takes less than 50 ms to reach the set value for flow rate. Moreover, the RSD of flow rate applied is lower than 5%. The rapidity and stability of controlling airflow rate with U-SAV allows creating a square-wave air flow profile. These validations comply with the AFNOR and ISO 20768 standard requirements.

### 4.5. AFNOR Recommendations

Both AFNOR and ISO recommendations have been verified and validated, with U-SAV meeting the requirements in terms of stability and repeatability. The power supply, air flow rate and mass variation of liquids A and B were tested and verified. All these parameters had RSDs below AFNOR and ISO standards recommendations. Thus, these results validate both the U-SAV machine and the Cubis atomiser for their utilization in research or for comparative studies and even for regulatory purposes.

## 5. Conclusions

We present in this study a novel vaping machine, U-SAV, which has the ability to generate, control and measure the electrical energy supplied to the resistor. The different experiments showed excellent reproducibility of the electrical parameters and of the applied airflow rate and an acceptable repeatability for the mass of aerosolised e-liquid. Our study demonstrates the importance of fully controlling the aerosolisation process. This work should be taken into consideration for the drafting of AFNOR, CEN and ISO standards. In order to have comparative results between laboratories, the batteries used for the tests must be well characterized and should be validated for their repeatability and consistent performance in order to be used in emission studies. Indeed, it was shown that even if a commercial battery device is reproducibly delivering power, there may be a significant difference between the power setting and the true power delivery.

It should be noted that the batteries used herein have a high capacity and can deliver power consistently over the 100 puffs required in the AFNOR standard. However, for batteries with smaller capacity or when higher power settings are used, the discharge rate will be faster and could affect the stability of the power supply during the experimental session. 

Because of its characteristics, U-SAV appears to be a suitable machine for standardized regulatory assessment of EC devices, Tobacco Products Directive (TPD) compliance and for research purposes. It can be used to study the influence of the main physical parameters of EC function such as power, resistor, puff number and duration, and how these parameters affect emissions. Indeed, the U-SAV interface allows changing several parameters, enabling the characterization of their influence on aerosol emissions. Moreover, U-SAV makes it possible to generate emissions with realistic vaper profiles such as sinusoidal air flow pattern or with the voltage mode that makes no adjustments relative to the change of the resistor value. U-SAV can also be used with commercial batteries and study their performance characteristics. Additionally, the power mode of USAV can be used to examine emissions under strictly controlled and accurate power delivery settings, to examine atomiser efficiency and to ensure inter-laboratory consistency in aerosol emission testing. 

## Figures and Tables

**Table 1:** Mass variation of aerosolized liquids A and B from 36 measurements per liquid (6 per Universal System for Analysis of Vaping (U-SAV) line) with the French association for standardization (AFNOR) standard protocol and a setting of 15 W in power mode, the standard deviation and the relative standard deviation (RSD).

Stability of the Mass of Consumed E-Liquid (15 W)			
	Average Consumption	Standard Deviation	RSD
**E-liquid A**	10.10 mg/puff	0.57 mg/puff	5.66%
**E-liquid B**	8.80 mg/puff	0.68 mg/puff	7.80%

**Table 2:** Mass variation of aerosolised liquids A and B, from 36 measurements per liquid (6 per U-SAV line) with a setting of 3.87 V.

Stability of the Mass of Consumed E-Liquid (3.87 V)			
	Average Consumption	Standard Deviation	RSD
**E-liquid A**	6.76 mg/puff	0.65 mg/puff	9.71%
**E-liquid B**	5.20 mg/puff	0,77 mg/puff	14.77%

**Table 3:** Power variation from 36 measurements (6 per U-SAV line) comparing energy delivery from U-SAV with two commercial EC battery devices.

Power Stability			
	Average Power Applied	Standard Deviation	RSD
**U-SAV**	14.68 W	0.06 W	0.44%
**Evic VTwo**	13.85 W	0.39 W	2.84%
**Elfin DNA40**	14.40 W	0.24 W	1.65%

**Table 4:** Mass variation of liquid A from 36 measurements (6 per U-SAV line) comparing energy delivery from U-SAV with two commercial electronic cigarette (EC) battery devices.

E-Liquid Mass Consumption Stability			
	Average Consumption	Standard Deviation	RSD
**U-SAV**	10.10 mg/puff	0.57 mg/puff	5.66%
**Evic VTwo**	8.55 mg/puff	0.40 mg/puff	4.63%
**Elfin DNA40**	9.62 mg/puff	0.84 mg/puff	8.75%

## References

[1] Farsalinos K.E., Polosa R. (2014). Safety evaluation and risk assessment of electronic cigarettes as tobacco cigarette substitutes: A systematic review. Ther. Adv. Drug Saf..

[2] Comte-Bellot G. (1976). Hot-wire anemometry. Annu. Rev. Fluid Mech..

[3] Lalo H., Soulet S., Casile C., Pairaud C. Correlation between Vapers Behaviour and Production of Degradation Products. Proceedings of the Poster Session Presented at the Global Forum Nicotine.

[4] AFNOR (2016). Cigarettes Electroniques et E-Liquides. Partie 3: Exigences et Méthodes D’essais Relatives aux Emissions. XP-D90–300.

[5] Talih S., Balhas Z., Eissenberg T., Salman R., Karaoghlanian N., El Hellani A., Shihadeh A. (2015). Effects of user puff topography, device voltage, and liquid nicotine concentration on electronic cigarette nicotine yield: Measurements and model predictions. Nicotine Tob. Res..

[6] Goniewicz M.L., Knysak J., Gawron M., Kosmider L., Sobczak A., Kurek J., Jacob P. (2014). Levels of selected carcinogens and toxicants in vapour from electronic cigarettes. Tob. Control.

[7] Kosmider L., Sobczak A., Fik M., Knysak J., Zaciera M., Kurek J., Goniewicz M.L. (2014). Carbonyl compounds in electronic cigarette vapors effects of nicotine solvent and battery output voltage. Nicotine Tob. Res..

[8] Farsalinos K.E., Voudris V., Poulas K. (2015). E-cigarettes generate high levels of aldehydes only in “dry puff” conditions. Addiction.

[9] Jensen R.P., Luo W., Pankow R.M., Strongin R.M., Peyton D.H. (2015). Hidden Formaldehyde in E-Cigarette Aerosols. New Eng. J. Med..

[10] McAuley T.R., Hopke P.K., Zhao J., Babaian S. (2012). Comparison of the effects of e-cigarette vapor and cigarette smoke on indoor air quality. Inhal. Toxicol..

[11] Goniewicz M.L., Kuma T., Gawron M., Knysak J., Kosmider L. (2013). Nicotine Levels in Electronic Cigarettes. Nicotine Tob. Res..

[12] Ingebrethsen B.J., Cole S.K., Alderman S.L. (2012). Electronic cigarette aerosol particle size distribution measurements. Inhal. Toxicol..

[13] Pourchez J., de Oliveira F., Perinel-Ragey S., Basset T., Vergnon J.M., Prévôt N. (2017). Assessment of new-generation high-power electronic nicotine delivery system as thermal aerosol generation device for inhaled bronchodilators. Int. J. Pharm..

[14] Callahan-Lyon P. (2014). Electronic cigarettes: Human health effects. Tob. Control.

[15] Farsalinos K.E., Romagna G., Allifranchini E., Ripamonti E., Bocchietto E., Todeschi S., Voudris V. (2013). Comparison of the cytotoxic potential of cigarette smoke and electronic cigarette vapour extract on cultured myocardial cells. Int. J. Environ. Res. Public Health.

[16] Goniewicz M.L., Hajek P., McRobbie H. (2013). Nicotine content of electronic cigarettes, its release in vapour and its consistency across batches: Regulatory implications. Addiction.

[17] Soulet S., Sorin J., Pairaud C., Mercury M., Lalo H. U-SAV Performance Data. Proceedings of the Poster Session Presented at the E-Cig Symposium.

[18] Manshadi M.D., Esfeh M.K. (2012). Analytical and Experimental Investigation About Heat Transfer of Hot-Wire Anemometry.

[19] Eguti C.C.A., Vieira E.D.R. Development of a Basic Circuit of a Hot-Wire Anemometer. Proceedings of the 10th Brazilian Congress of Thermal Sciences and Engineering.

[20] Ferreira R.P.C., Freire R.C.S., Deep C.S., de Rocha Neto J.S., Oliveira A. (2001). Hot-wire anemometer with temperature compensation using only one sensor. IEEE Trans. Instrum. Meas..

[21] Lide D.R. (2007). CRC Handbook of Chemistry and Physics.

